# ﻿The genus *Hercostomus* Loew (Diptera, Dolichopodidae, Dolichopodinae) from Inner Mongolia, China, with the description of two new species

**DOI:** 10.3897/zookeys.1118.84403

**Published:** 2022-08-24

**Authors:** Xingyang Qian, Ning Wang, Ding Yang

**Affiliations:** 1 Institute of Grassland Research, Chinese Academy of Agricultural Sciences, Hohhot, Inner Mongolia 010010, China Institute of Grassland Research, Chinese Academy of Agricultural Sciences Hohhot China; 2 Department of Entomology, College of Plant Protection, China Agricultural University, Beijing 100193, China China Agriculture University Beijing China

**Keywords:** Identification key, long-legged flies, new records, taxonomy

## Abstract

Previously, only two species of *Hercostomus* Loew were known to occur in Inner Mongolia. Here two species from Inner Mongolia are described as new to science, namely *Hercostomuschifengensis***sp. nov.** and *Hercostomustriangulatus***sp. nov.** Three new records of *Hercostomus* in Inner Mongolia are added. A key to the species of *Hercostomus* in Inner Mongolia is provided.

## ﻿Introduction

*Hercostomus* Loew is one of the largest genera in the family Dolichopodidae with 475 known species worldwide, of which 300 species have been recorded from China (Yang et al. 2006; Yang et al. 2011; Qilemoge et al. 2017, 2020; Grichanov 2020). Members of *Hercostomus* can be identified by the following features: eyes separated at the lower margin; thorax lacking a distinct dark spot above the notopleuron, pleural surface in front of the posterior spiracle bare; mid femora with an anterior preapical bristle; hind femora with the anterior bristle positioned at the apex, usually slightly flattened laterally, or not; fore tarsus usually simple; wing rarely darkened in the anterior half; vein M_1+2_ weakly sinuate, flexion at the basal third or at the middle of the distal part and sometimes with subapical flexion; sometimes the basiventral epandrial lobe of the epandrium and hypandrium forming complex entangled asymmetrical lobes (Brooks 2005; Grichanov 2011; Yang et al. 2011).

Inner Mongolia is located in a narrow region extending northeast to southwest in northern China. The climate of Inner Mongolia is temperate continental with greater precipitation in the northeast compared to the southwest and higher temperatures in the southwest compared to the northeast. Natural vegetation types range from forests, meadow steppe, typical steppe, desert steppe and the Gobi Desert from the northeast to the southwest, respectively.

**Figure 1.**
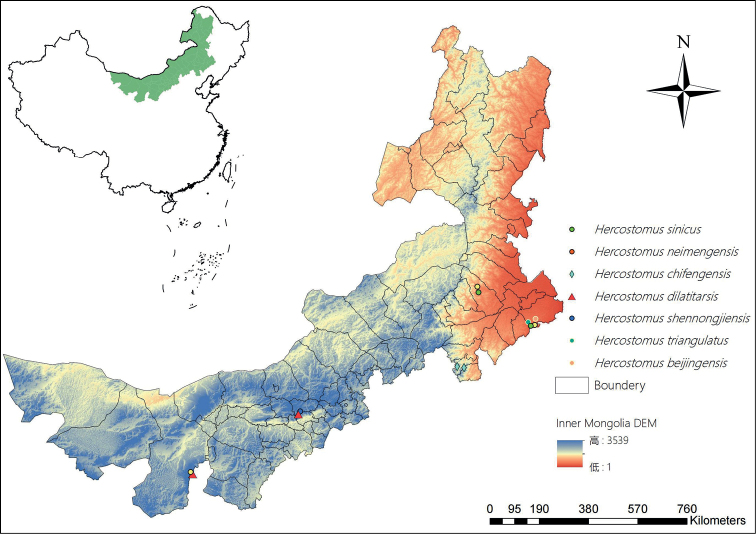
Distribution of *Hercostomus* in Inner Mongolia.

Previously, only two species, *Hercostomusneimengensis* Yang, 1997 and *H.sinicus* Stackelberg, 1934, were recorded from Inner Mongolia (Yang et al. 2011). Here two new species of *Hercostomus* are described from Inner Mongolia, namely *H.chifengensis* sp. nov. and *H.triangulatus* sp. nov. The following three species are newly recorded from Inner Mongolia: *Hercostomusbeijingensis* Yang, 1996, *Hercostomusdilatitarsis* Stackelberg, 1949 and *Hercostomusshennongjiensis* Yang, 1997. A key to species of *Hercostomus* in Inner Mongolia is provided. All of the updated records are distributed in mountains of nature reserves in Inner Mongolia: Jiufeng Mountain, Helan Mountain, Daqinggou. *Hercostomusneimengensis* Yang, 1997 is also distributed in grasslands of Keerqin. We discovered that all *Hercostomus* species in Inner Mongolia are distributed in grasslands near creeks and damp areas of mountains (Fig. 1). Currently, the genus comprises 300 species in China and is distributed widely around China. The low level of diversity of the genus in Inner Mongolia is probably the result of few investigations (Yang et al. 2011). Thus, it is promising to find more *Hercostomus* species in Inner Mongolia, especially in forests of northeast part of Inner Mongolia.

## ﻿Materials and methods

The specimens on which this study is based were collected from Inner Mongolia in 2013 and 2014 by sweeping net. All specimens are deposited in the Entomological Museum of China Agricultural University (CAU), Beijing. Morphological terminology follows Cumming and Wood (2017). The following abbreviations are used: **acr** = acrostichal bristle (s), **ad** = anterodorsal bristle (s), **av** = anteroventral bristle (s), **dc** = dorsocentral bristle (s), **sc** = scutellars, **pd** = posterodorsal bristle (s), **v** = ventral bristle (s), **LI** = fore leg, LII = mid leg, **LIII** = hind leg, **CuAx ratio** = length of dm–cu / length of distal portion of CuA.

## ﻿Taxonomy

### ﻿Key to species (males) of *Hercostomus* from Inner Mongolia

**Table:** 

1	Antenna entirely black	**2**
–	Antenna yellow or partly dark yellow	**4**
2	Abdominal tergites (Figs 3, 7) wholly metallic green; male cercus (Figs 10, 11) with distinct denticles	**3**
–	Abdominal tergites 1–3 (Fig. 2) yellow at lateral margin; male cercus with indistinct or weak denticles	***H.beijingensis* Yang**
3	Postpedicel (Fig. 9) 1.8 times longer than wide, blunt at tip; male cercus (Fig. 10) lobate, slightly shorter than epandrium, distinctly longer than wide, with several short finger-like marginal processes	***H.chifengensis* sp. nov.**
–	Postpedicel 1.3 times longer than wide, sharp at tip; male cercus (Fig. 11) long strip-like, geniculate, apical half with long marginal bristles hook-like apically	***H.sinicus* Stackelberg**
4	Epandrial lobe very long finger-like; male cercus very narrow, long strip-like with indistinct or weak digitations	***H.shennongjiensis* Yang**
–	Epandrial lobe (Fig. 14) very short or absent; male cercus (Fig. 14) rather wide, somewhat quadrate or triangular with distinct digitations	**5**
5	Fore tarsomeres 2–5 flattened; epandrium slightly longer than wide, with nearly truncate apical margin	***H.dilatitarsis* Stackelberg**
–	Fore tarsus simple; epandrium (Fig. 14) distinctly longer than wide, with convex apical margin	**6**
6	Postpedicel blackish at base, obtuse at tip; male cercus band-like with some marginal denticles at tip	***H.neimengensis* Yang**
–	Postpedicel (Fig. 12) blackish with basal ventral surface dark yellow, acute at tip; male cercus (Fig. 14) nearly triangular with weak denticles and 3 relatively long finger-like processes	***H.triangulatus* sp. nov.**

#### ﻿ Hercostomus beijingensis

Taxon classificationAnimaliaDipteraDolichopodidae

Yang, 1996

DC425294-F60D-5C3B-B060-0216B436F030

Fig. 2

Hercostomus beijingensis Yang, 1996: 318. Type locality: China: Beijing, Yingtaogou.

##### Diagnosis.

Antenna entirely black; postpedicel 1.8 times longer than wide, blunt at tip. Metapleuron yellow. Abdominal tergites 1–3 yellow at lateral margin. All coxae entirely yellow. Male cercus nearly quadrate. Phallus thin and long, apically geniculate.

**Figures 2–5.**
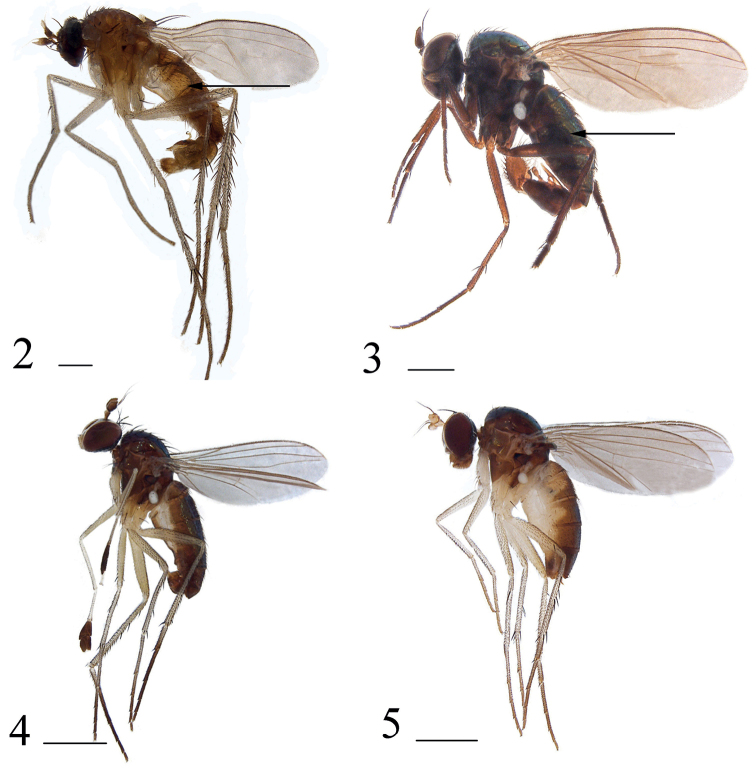
Habitus, lateral view **2***Hercostomusbeijingensis* Yang, 1966, male **3***Hercostomuschifengensis* sp. nov., holotype male **4***Hercostomusdilatitarsis* Stackelberg, 1949, male **5***Hercostomusneimengensis* Yang, 1997, female. Scale bars: 1 mm.

##### Specimens examined.

***Holotype***: male, China, Beijing, Xiangshan, Yingtaogou, 1987.V.30, Ding Yang (CAU). **Other material**: 2 males, China, Inner Mongolia, Tongliao, Daqinggou, 200–300m, 2014.VII.22, Ning Wang & Ding Yang (CAU).

##### Distribution.

China (Inner Mongolia, Beijing, Henan, Shanxi, Hubei).

#### ﻿ Hercostomus chifengensis sp. nov.

Taxon classificationAnimaliaDipteraDolichopodidae

4925BE34-7207-5470-A669-E615446A0FD4

https://zoobank.org/BAA059A0-E3CE-4AF2-AEDD-61689F5F5270

Figs 3 , 9–10

##### Diagnosis.

Antenna entirely black; postpedicel 1.8 times longer than wide, blunt at tip; basal segment of arista 0.55 times as long as apical segment. Legs entirely black. Wings slightly tinged brown. Male cercus nearly lobate, distinctly longer than wide, with short finger-like marginal processes.

**Figures 6–8.**
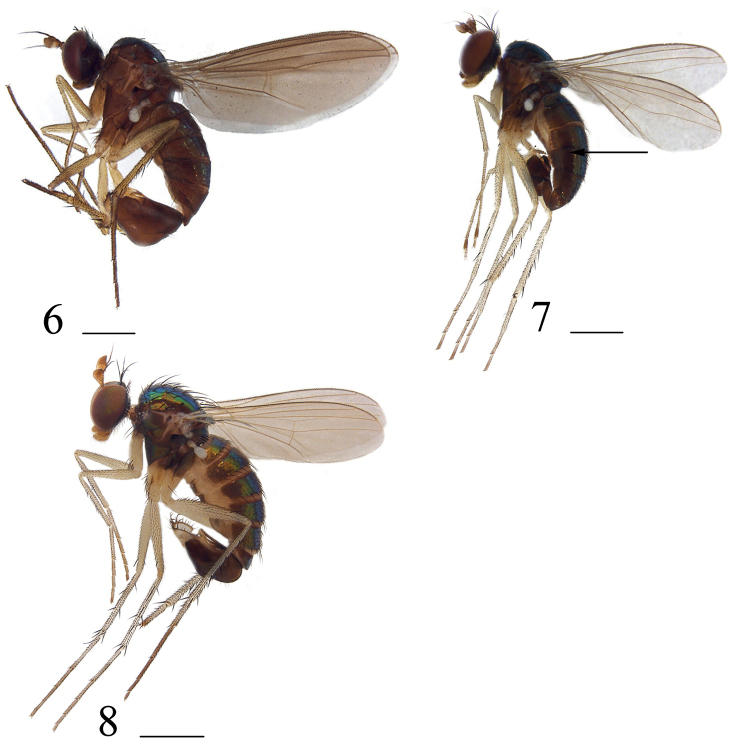
Habitus, lateral view **6***Hercostomusshennongjiensis* Yang, 1997, male **7***Hercostomussinicus* Stackelberg, 1934, male **8***Hercostomustriangulatus* sp. nov., holotype male. Scale bars: 1 mm.

##### Description.

**Male** (Fig. 3). Body length 3.1–3.2 mm, wing length 3.5–4.1 mm.

**Figures 9, 10.**
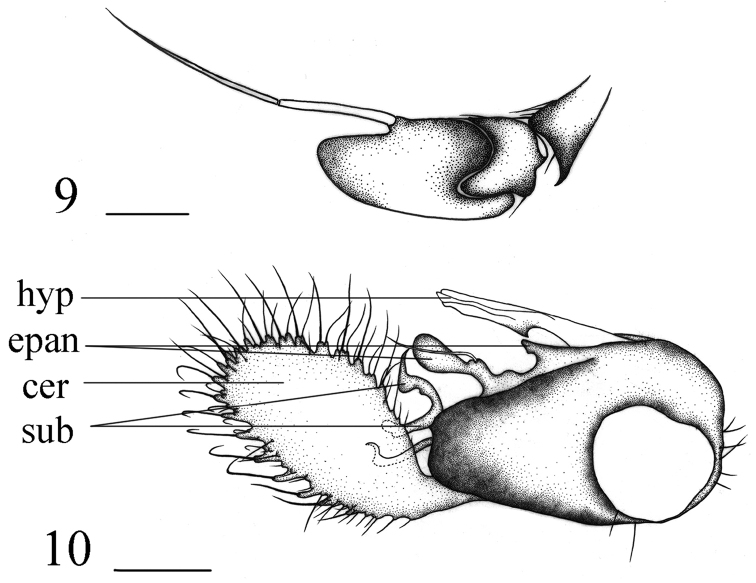
*Hercostomuschifengensis* sp. nov., male. **9** antenna, lateral view **10** genitalia, lateral view. Abbreviations: hyp = hypandrium, epan = epandrial lobe, cer = cercus, sub = subepandrial process. Scale bars: 0.1 mm.

***Head*** metallic green with pale grey pollinosity. Hairs and bristles on head black, but middle and lower postocular bristles and posteroventral hairs yellow. Ocellar tubercle with 2 strong oc and 2 short posterior hairs. Antenna (Fig. 9) black; postpedicel 1.8 times longer than wide, blunt at tip; arista black, basal segment 0.55 times as long as apical segment. Proboscis brownish with black hairs; palpus black with black hairs and 1 black apical bristle.

***Thorax*** metallic green with pale grey pollinosity. Hairs and bristles on thorax black; 7–8 irregularly biseriate acr short hair-like, 6 long strong dc. Scutellum with 2 pairs of sc and several short marginal hairs, basal pair hair-like.

***Legs*** entirely black. Hairs and bristles on legs black. Mid and hind coxae each with 1 outer bristle; mid and hind femora each with 1 preapical bristle; fore tibia with 3 short ad, 2 short pd and 2 apical bristles; mid tibia with 4 ad, 2 pd, 1 av and 3 apical bristles; hind tibia with 3 ad, 2 pd, 1 short av and 3 apical bristles; hind tarsomere 1 with 1 short v at base. Relative lengths of tibia and 5 tarsomeres of legs LI: 1.8: 0.8: 0.4: 0.3: 0.2: 0.2; LII: 2.7: 1.15: 0.6: 0.5: 0.3: 0.2; LIII: 3.4: 0.85: 1.0: 0.7: 0.45: 0.3. ***Wing*** nearly hyaline, slightly tinged brownish; veins brown; R_4+5_ and M_1+2_ distinctly convergent apically; CuAx ratio 0.55. Squama yellow with blackish hairs. Halter yellow.

***Abdomen*** metallic green with pale grey pollinosity. Hairs and bristles on abdomen black. Male genitalia (Fig. 10): Epandrium distinctly longer than wide, narrowed at tip; inner epandrial lobe relatively small, outer epandrial lobe long finger-like with somewhat swollen tip. Subepandrial process with two long processes branched, one blunt at tip, one sharp at tip. Male cercus large, lobate, slightly shorter than epandrium, distinctly longer than wide, with several short finger-like marginal processes. Hypandrium tubular at tip, with a hook-like projection near middle.

**Female**. Unknown

##### Type material examined.

***Holotype***: male, China, Inner Mongolia, Chifeng, Wangyedian, Binlanggoumen, 1223 m, 2014.VIII.25, Li Shi (CAU). ***Paratype***: 1 male, China, Inner Mongolia, Chifeng, Saihanwula, 1200 m, 2013.VII.24, Xiumei Lu (CAU).

##### Distribution.

China (Inner Mongolia).

##### Remarks.

The new species is somewhat similar to *H.subrusticus* Zhang, Yang & Grootaert, 2008 from Xinjiang of China, but can be distinguished from the latter by the arista located at middle of the dorsal margin of the postpedicel and the male cercus long and narrow. In *H.subrusticus*, the arista is located at the apical one-third of the dorsal margin of the postpedicel, and the male cercus is relatively short and wide (Zhang et al. 2008).

##### Etymology.

The species is named after the type locality Chifeng.

#### ﻿ Hercostomus dilatitarsis

Taxon classificationAnimaliaDipteraDolichopodidae

Stackelberg, 1949

1C5AE146-5BB8-515E-AB00-EEAA7A3807CD

Fig. 4

Hercostomus dilatitarsis Stackelberg, 1949: 687. Type locality: Tajikistan: Kondara. Valley Varzob. Gissar Ridge.

##### Diagnosis.

Postpedicel entirely black. Coxae entirely yellow, but mid coxa tinged blackish. Fore tarsomere 1 yellow, tarsomeres 2–3 distinctly flattened and black, tarsomeres 4–5 weakly flattened and white.

##### Specimens examined.

1 male, China, Inner Mongolia, Mount Jiufeng, Toudaogou, 1500–1600 m, 2013.VIII.4, Xiao Zhang (CAU). 3 males 3 females, China, Inner Mongolia, Helan Mountain, Shuimogou, 1800–1900 m, 2010.VIII.6, Lihua Wang (CAU).

##### Distribution.

China (Inner Mongolia, Hebei); Tajikistan.

#### ﻿ Hercostomus neimengensis

Taxon classificationAnimaliaDipteraDolichopodidae

Yang, 1997

A95DBA86-6DEF-54E8-9751-81F30EDEEDD0

Fig. 5

Hercostomus (Hercostomus) neimengensis Yang, 1997: 138. Type locality: China: Inner Mongolia, Tuyouqi.

##### Diagnosis.

Antenna yellow with postpedicel blackish at tip and 1.1 times longer than wide. Thorax metallic green, except hypopleuron partly yellow and metapleuron entirely yellow. Legs including coxae yellow; hairs and bristles on coxae yellowish. Male cercus band-like with some marginal denticles at tip.

##### Specimens examined.

***Holotype***: male, China, Inner Mongolia, Tumoteyouqi, 1978.VII.21, Heming Chen (CAU). **Other material**: 1 male 8 females, China, Inner Mongolia, Helan Mountain, Xiangchizigou, 1900 m, 2013.VII.30, Xiao Zhang (CAU).

##### Distribution.

China (Inner Mongolia, Gansu).

#### ﻿ Hercostomus shennongjiensis

Taxon classificationAnimaliaDipteraDolichopodidae

Yang, 1997

78FF9F24-B1A0-52D9-9FC4-208E6A0C9CF5

Fig. 6

Hercostomus (Hercostomus) shennongjiensis Yang, 1997: 118. Type locality: China: Hubei, Shennongjia.

##### Diagnosis.

Postpedicel dark yellow at basal ventral portion, 1.2 times longer than wide, somewhat acute at tip. All coxae entirely black or blackish. Wing slightly brownish. Male cercus long strip-like with short hairs. Epandrial lobe long finger-like with very long apical bristles.

##### Specimens examined.

1 male 1 female, China, Inner Mongolia, Mount Jiufeng, Erdaogou, 1400–1500 m, 2013.VIII.3, Xiumei Lu (CAU).

##### Distribution.

China (Inner Mongolia, Hubei, Shanxi, Henan).

#### ﻿ Hercostomus sinicus

Taxon classificationAnimaliaDipteraDolichopodidae

Stackelberg, 1934

6CBD4D07-5576-5F85-BAAC-06AB3ED5B7C3

Figs 7 , 11

Hercostomus sinicus Stackelberg, 1934: 174. Type locality: China: “Dyn-uan-in, Nord-Alashan”.

##### Diagnosis.

Postpedicel 1.3 times longer than wide, sharp at tip. Fore tarsomeres 1–3 relatively thin, tarsomeres 4–5 weakly thickened, tarsomere 4 dark brown, tarsomere 5 white. Male cercus long strip-like, geniculate, apical half with long marginal bristles hook-like apically.

##### Description.

**Male** (Fig. 7). Body length 3.1–3.2 mm, wing length 3.0–3.2 mm.

***Head*** metallic green with pale grey pollinosity. Hairs and bristles on head black, but middle and lower postocular bristles and posteroventral hairs yellow. Antenna black; postpedicel nearly square, 1.3 times longer than wide, sharp at tip; arista black with short hairs, basal segment 0.2 times as long as apical segment. Proboscis brownish yellow with brownish yellow hairs; palpus blackish with brownish yellow hairs and 1 blackish apical bristle.

***Thorax*** metallic green with pale grey pollinosity. Hairs and bristles on thorax black; 6 irregularly biseriate acr slightly long and strong, 6 long strong dc. Scutellum with 2 pairs of sc, basal pair hair-like. Propleuron with yellowish hairs and 1 bristle on lower portion.

***Legs*** mostly yellow. Fore coxa yellow, mid coxa blackish, hind coxa brownish yellow; fore tarsomere 4 dark brown, tarsomere 5 white; mid and hind tarsus brown or dark brown from tip of tarsomere 1 onwards. Fore tarsomeres 1–3 relatively thin, tarsomeres 4–5 weakly thickened. Hairs and bristles on legs black, but some hairs and bristles on coxae yellow; mid and hind coxae each with 1 outer bristle; mid and hind femora each with 1 preapical bristle. Fore tibia with 1 ad, 2 pd and 3 apical bristles (apico-ventral bristle brown, 1/4 as long as tarsomere 1); mid tibia with 2–3 ad, 2 pd and 4 apical bristles; hind tibia with 2 ad, 3 pd and 4 apical bristles (including 1 subapical pd). Relative lengths of tibia and 5 tarsomeres of legs LI: 2.25: 1.1: 0.85: 0.6: 0.35: 0.3; LII: 2.75: 1.45: 0.8: 0.65: 0.4: 0.3; LIII: 3.2: 0.9: 1.2: 0.7: 0.5: 0.3. ***Wing*** nearly hyaline, veins dark brown; R_4+5_ and M_1+2_ distinctly convergent apically; CuAx ratio 0.4. Squama yellow with brown hairs. Halter yellow.

**Figure 11.**
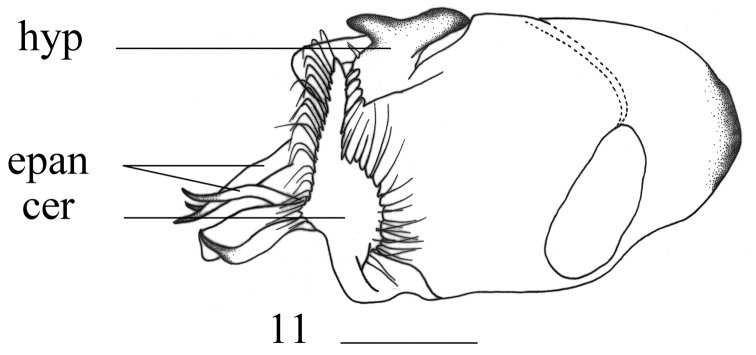
*Hercostomussinicus* Stackelberg, 1934, male genitalia, lateral view. Abbreviations: hyp = hypandrium, epan = epandrial lobe, cer = cercus. Scale bars: 0.1 mm.

***Abdomen*** metallic green with pale grey pollinosity except hypogygium brownish yellow at tip. Hairs and bristles on abdomen black. Male genitalia (Fig. 11): Epandrium distinctly longer than wide; epandrial lobe weakly bulged. Male cercus long strip-like, geniculate, apical half with long marginal bristles hook-like apically. Hypandrium irregularly branched, right process short, left process long and hook-like.

**Female**. Body length 3.2–3.5 mm, wing length 3.0–3.2 mm.

##### Specimens examined.

2 males, China, Inner Mongolia, Tongliao, Daqinggou, 180 m, 2014.VII.22, Ning Wang & Ding Yang (CAU).

##### Distribution.

China (Inner Mongolia).

##### Remarks.

This species is redescribed with illustrations of male genitalia for the first time.

#### ﻿ Hercostomus triangulatus sp. nov.

Taxon classificationAnimaliaDipteraDolichopodidae

A9544386-B1C6-53EC-A4D1-A61A1E4228FE

https://zoobank.org/76823345-6706-4FC3-BBAF-D3087E4E34C4

Figs 8 , 12–14

##### Diagnosis.

Antenna mainly dark yellow; postpedicel blackish with basal ventral surface dark yellow, 1.2 times longer than wide, obtuse at tip; arista black, basal segment 0.25 times as long as apical segment. All coxae dark yellow. Male cercus nearly triangular with weak denticles and 3 relatively long finger-like processes.

##### Description.

**Male** (Fig. 8). Body length 3.7–4.1 mm, wing length 3.3–3.6 mm.

**Figures 12–14.**
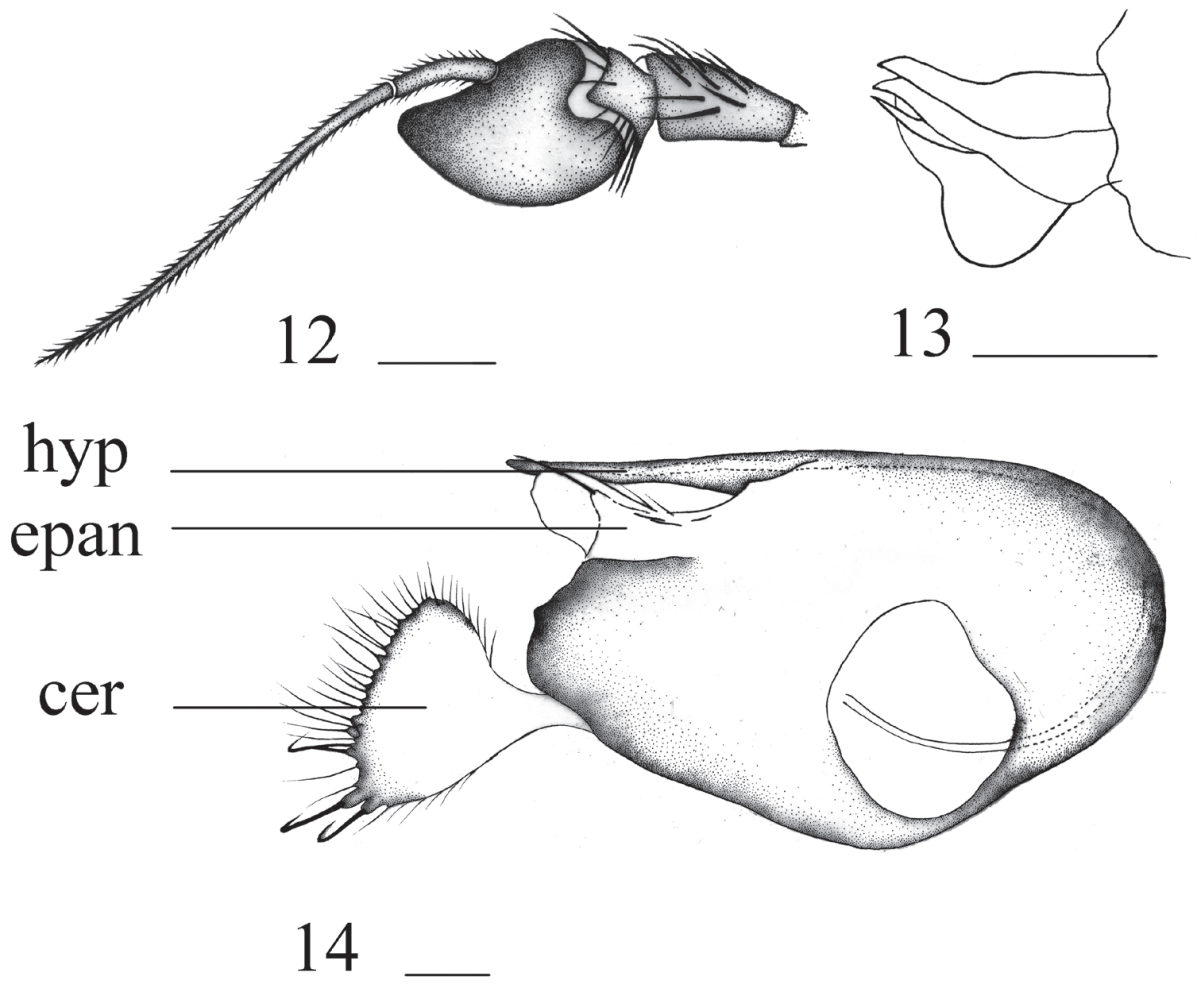
*Hercostomustriangulatus* sp. nov., male **12** antenna, lateral view **13** subepandrial processess and postgonite, lateral view **14** genitalia, lateral view. Abbreviations: hyp = hypandrium, epan = epandrial lobe, cer = cercus. Scale bars: 0.1 mm.

***Head*** metallic green with dense pale grey pollinosity. Hairs and bristles on head black, middle and lower postocular bristles and posteroventral hairs yellow. Antenna (Fig. 12) dark yellow except scape blackish at base and postpedicel blackish with base and ventral surface dark yellow; postpedicel 1.2 times longer than wide, somewhat acute at tip; arista blackish with short pubescence, basal segment 0.25 times as long as apical segment. Proboscis brownish yellow with black hairs; palpus brownish, with dark yellow hairs and 1 dark yellow apical bristle.

***Thorax*** metallic green with pale grey pollinosity. Hairs and bristles on thorax black; 6~8 irregularly biseriate acr short hair-like; 6 long strong dc. Scutellum with 2 pairs of sc, basal pair short hair-like. Propleuron with yellowish hairs and 1 black bristle on lower portion.

***Legs*** yellow; all coxae yellow; all tarsi brown to dark brown from tip of tarsomere 1 onwards. Hairs and bristles on legs black; mid and hind coxae each with 1 outer bristle; mid and hind femora each with 1 preapical bristle; fore tibia with 1 ad, 2 pd and 2 short apical bristles; mid tibia with 4 ad, 2 short pd, 1 av and 4 short apical bristles; hind tibia with 3 ad, 3 pd, 4 short av (2 inner bristles thin, 2 outer bristles thick) and 3 apical bristles. Hind tarsomere 1 with 1 short ventral bristle at base. Relative lengths of tibia and 5 tarsomeres of legs LI: 2.0: 1.1: 0.5: 0.4: 0.3: 0.2; LII: 2.8: 1.5: 0.8: 0.7: 0.4: 0.3; LIII: 3.4: 1.0: 1.2: 0.5: ?: ?. ***Wing*** nearly hyaline, veins dark brown; R_4+5_ and M distinctly convergent apically; CuAx 0.5. Squama yellow with dark yellowish hairs. Halter yellow.

***Abdomen*** metallic green with pale grey pollinosity. Hairs and bristles on abdomen black; tergite 1 with several short yellow hairs; sternites 2–3 with short yellow hairs. Male genitalia (Fig. 14): Epandrium distinctly longer than wide, narrowed at tip; epandrial lateral lobe relatively short and thick. Subepandrial process (Fig. 13) with two processes separated, narrowed at tip. Male cercus nearly triangular with some weak denticles and 3 relatively long finger-like processes bearing long bristles on apical margin. Hypandrium somewhat acute at tip.

**Female.** Body length 3.0–3.6 mm, wing length 3.6–3.7 mm.

##### Type material examined.

***Holotype***: male, China, Inner Mongolia, Tongliao, Daqinggou, 180m, 2014.VII.24, Ning Wang & Ding Yang (CAU). ***Paratypes***: 3 males 1 female, same data as holotype (CAU); 8 males 3 females, China, Inner Mongolia, Tongliao, Daqinggou, 180 m, 2014.VII.23, Ning Wang & Ding Yang (CAU); 1 male 1 female, China, Tongliao, Daqinggou, 180 m, 2014.VII.22, Ning Wang & Ding Yang (CAU).

##### Distribution.

China (Inner Mongolia).

##### Remarks.

The new species is somewhat similar to the members of *H.crassivena* group, but the veins of *H.triangulatus* are not thickened (Zhang and Yang 2007).

##### Etymology.

This species is named after the triangular cercus.

## ﻿Discussion

*Hercostomus* Loew is probably polyphyletic and not a monophyletic genus as it is poorly defined (Brooks 2005; Yang et al. 2011). Currently 300 species of *Hercostomus* are distributed in China, of which seven species are distributed in Inner Mongolia. Twenty-four species groups of *Hercostomus* distributed in China were recognized (Yang et al. 2011; Grichanov 2020), namely *H.crassivena* group, *H.abnormis* group, *H.longicercus* group, *H.quadriseta* group, *H.takagii* group, *H.fatuus* group, *H.ulrichi* group, *H.flavimaculatus* group, *H.subnovus* group, *H.flaviventris* group, *H.curvus* group, *H.albidipes* group, *H.apiculatus* group, *H.baishanzuensis* group, *H.nanlingensis* group, *H.absimilis* group, *H.intactus* group, *H.longus* group, *H.fluvius* group, *H.prolongatus* group, *H.digitiformis* group, *H.biancistrus* group, *H.incisus* group, *H.digitatus* group. As to the seven species distributed in Inner Mongolia, *H.shennongjiensis* Yang, 1997 belongs to the *H.digitiformis* group, *H.chifengensis* sp. nov. belongs to the *H.nanlingensis* group, and *H.beijingensis* Yang, 1996 belongs to the *Hercostomussubnovus* group, while *H.dilatitarsis* Stackelberg, 1949, *H.neimengensis* Yang, 1997, *H.sinicus* Stackelberg, 1934, and *H.triangulatus* sp. nov. were not assigned to any species group. Further studies are necessary in order to clarify their systematic placement.

## Supplementary Material

XML Treatment for Hercostomus beijingensis

XML Treatment for Hercostomus chifengensis

XML Treatment for Hercostomus dilatitarsis

XML Treatment for Hercostomus neimengensis

XML Treatment for Hercostomus shennongjiensis

XML Treatment for Hercostomus sinicus

XML Treatment for Hercostomus triangulatus
